# Cannabinoids and PPAR Ligands: The Future in Treatment of Polycystic Ovary Syndrome Women with Obesity and Reduced Fertility

**DOI:** 10.3390/cells11162569

**Published:** 2022-08-18

**Authors:** Piotr Przybycień, Danuta Gąsior-Perczak, Wojciech Placha

**Affiliations:** 1 Chair of Medical Biochemistry, Faculty of Medicine, Jagiellonian University Medical College, 31-034 Krakow, Poland; 2 Endocrinology Clinic, Holycross Cancer Centre, 25-734 Kielce, Poland; 3 Collegium Medicum, Jan Kochanowski University, 25-317 Kielce, Poland

**Keywords:** CB1R, CB2R, PCOS, PPARs, cannabinoids, endocannabinoids, obesity, infertility, cannabis, THC

## Abstract

Cannabinoids (CBs) are used to treat chronic pain, chemotherapy-induced nausea and vomiting, and multiple sclerosis spasticity. Recently, the medicinal use of CBs has attracted increasing interest as a new therapeutic in many diseases. Data indicate a correlation between CBs and PPARs via diverse mechanisms. Both the endocannabinoid system (ECS) and peroxisome proliferator-activated receptors (PPARs) may play a significant role in PCOS and PCOS related disorders, especially in disturbances of glucose-lipid metabolism as well as in obesity and fertility. Taking into consideration the ubiquity of PCOS in the human population, it seems indispensable to search for new potential therapeutic targets for this condition. The aim of this review is to examine the relationship between metabolic disturbances and obesity in PCOS pathology. We discuss current and future therapeutic interventions for PCOS and related disorders, with emphasis on the metabolic pathways related to PCOS pathophysiology. The link between the ECS and PPARs is a promising new target for PCOS, and we examine this relationship in depth.

## 1. Introduction

Approximately 10% of women (6.1 million) in the United States of America between the ages of 15 and 44 have difficulty conceiving or carrying pregnancies [1]. Being overweight or obese is well-known to compromise reproductive health. Women with BMI ≥ 25 kg/m^2^ have a significantly higher chance of miscarriage [2]. The majority of patients with diagnosed PCOS are overweight or obese (40–70%) [3,4,5]. The prevalence of PCOS in reproductive-age women in the United States (US) ranges from 6% to 12% (as many as 5 million) [6]. PCOS is the most common endocrine disorder among women of reproductive age [7], and is linked to multiple conditions such as metabolic syndrome, obesity, impaired glucose tolerance, diabetes mellitus type 2, cardiovascular disease, non-alcoholic fatty liver disease/ non-alcoholic steatohepatitis (NAFLD/NASH), and infertility. The pathophysiology of PCOS is complex and remains incompletely understood, and the present review will summarize known contributors to PCOS and suggest potential additional mechanisms of action.

Among women with PCOS, obesity and overweight are common findings, and moreover, they are a problem in themselves for women’s health. Over the past few decades, the incidence of obesity has increased exponentially, and many non-obese individuals are overweight. The World Health Organization (WHO) officially declared obesity an epidemic in 1997 [8]. Data obtained in 2016 estimate the total number of overweight persons as more than 1.9 billion adults, of which 650 million are obese [9].

The endocannabinoid system is widespread in the human body. The ECS consists of cannabinoid receptors (CBRs), their ligands, and enzymes regulating their biosynthesis and biodegradation [10]. This ECS is involved in cognitive processes, appetite regulation, vomiting, motor skill regulation, and neuroendocrine systems. It also modulates energy metabolism and the immune response [11]. Energy metabolism is regulated by appetite, food intake, and energy disposition. These processes are regulated by the nucleus accumbens, hypothalamus, muscles, digestive tract, liver, and adipose tissue. Marijuana is the most commonly used form of cannabis worldwide. It contains diverse CBs, for example, the psychoactive cannabinoid Δ-9-THC, and short-term (13 day) marijuana consumption increases appetite, food intake, and body weight in healthy male users [12]. Cannabinoid receptor type 1 (CB1R) is expressed on appetite-related structures of the rat brain [13]. Components of the ECS are expressed in peripheral organs that regulate metabolic homeostasis, such as adipose tissue, pancreas, liver, skeletal muscles, and certain elements of the digestive tract. The ECS is involved in fertility and reproduction in humans and influences both the female and male reproductive systems [14]. In the male reproductive system, CBs and their receptors are present in Sertoli cells and Leydig cells in the testes and in sperm cells in various species ranging from invertebrates to mammals [15]. Elements of the ECS are present in female reproductive organs such as the follicles, ovaries, oviduct, and uterus and influence gametogenesis, fertilization, and embryo implantation [16,17,18,19,20,21,22]. The available data indicate that there is an association between the ESC and the PPARs.

PPARs regulate crucial processes of cellular energy metabolism, cell proliferation, and inflammation. PPARs belong to a superfamily of nuclear receptor proteins consisting of transcription factors that regulate transcription of genes involved in multiple processes such as glucose and lipid metabolism, and altered PPAR expression is potentially correlated with diseases such as dyslipidemia, obesity, metabolic syndrome, and type 2 diabetes mellitus (DM-2) [23,24]. PPARs regulate the balance between anabolic and oxidative processes, and as a result, control adipose tissue homeostasis [24]. There are three PPAR types, which differ in tissue distribution, ligand affinity, and biological function [25]. The most common PPAR ligands are fatty acids and fatty-acid-derived eicosanoids. In clinical practice, synthetic PPAR ligands such fibrates are used to treat dyslipidemia, and thiazolidinediones (glitazones) are used to treat hyperglycemia.

Many prior findings suggest a role for CBs and PPARs in the pathophysiology of obesity and related morbidities such as PCOS. Some PPAR agonists such as fibrates and thiazolidinediones are currently used in clinical practice in treatment of hypertriglyceridemia and DM-2, respectively. Additionally, thiazolidinediones are also used to treat PCOS. Cannabinoid ligands have been considered as potential obesity treatments [26]. Moreover, the ECS is linked with PPARs, and CBs are thus a potential therapy for PCOS.

This systematic review is divided into three sections. First, we will focus on the pathophysiology of PCOS, obesity, and infertility. Secondly, we will review the structure and function of the ECS and each of the PPAR types (α, β/δ, and γ), with particular emphasis on their influences on energy metabolism and fertility. Finally, we will discuss relationships between the ECS and PPARs. We will examine the evidence that cannabinoids, cannabinoid-like compounds, and their metabolites activate PPARs, and discuss non-cannabinoid dual *CBRs*/PPAR ligands. We discuss other commonalities between the CBs and PPAR pathways. In the end, we will summarize the evidence and draw a conclusion.

## 2. PCOS

PCOS is a complex endocrine disorder and is diagnosed when differential diagnoses such as thyroid disease, hyperprolactinemia, and non-classical congenital adrenal hyperplasia have been excluded [7]. The 2003 Rotterdam criteria are used to diagnose PCOS, and two of the following three criteria are required: (1) oligo- or anovulation; (2) clinically or biochemically verified hyperandrogenism; and (3) ovarian polycystic morphology revealed by ultrasound scan and meeting the following criteria: 12 or more ovarian follicles and ovary diameter 2–9 cm and/or enlarged ovarian volume >10 mL, calculated as 0.5 × length × width [27]. In 2018, updated international evidence-based recommendations for assessment and management of PCOS were announced. The guidelines encompass a wide range of PCOS criteria. For example, the 2018 guideline raised the antral follicle number threshold for PCOS diagnosis from 12 to 20 with the use of a high-frequency probe [28]. The National Institutes of Health Consensus Conference in 2012 recommended use of the 2003 Rotterdam criteria, but on the condition that specific PCOS phenotypes are diagnosed [29]. There are four recognized phenotypes of PCOS [29], the criteria of which are shown in Table 1 [30].

PCOS phenotypes are associated with different degrees of metabolic dysfunction, long-term health problems, and different treatment approaches. For example, patients with PCOS phenotype A have higher menstrual irregularities, ovarian reserve parameters, BMI, hyperandrogenism (clinical and biochemical), fasting insulin, and dyslipidemia [30]. PCOS is associated with many morbidities, including metabolic syndrome, obesity, impaired glucose tolerance, DM-2, NAFLD/NASH, and infertility [7]. The etiology of PCOS remains incompletely understood. The pathophysiology of PCOS is complex, with strong epigenetic and environmental influences, including diet and lifestyle.

### 2.1. PCOS and Hormone Imbalance

One of the most important characteristics in women with PCOS is hyperandrogenism, underscoring its pathological role in the development and progression of PCOS. The prevalence of hyperandrogenemia among women with PCOS (Rotterdam criteria) is 58.8% [31]. Hirsutism is the most common clinical feature of hyperandrogenism. In the female body, the primary sources of androgens are the zona reticularis of the adrenal gland (ACTH-regulated adrenal androgen) and theca cells in the ovaries (LH-regulated ovarian androgen) [32]. Small amounts of androgens are produced in peripheral tissues [32]. Excessive androgen production in the ovaries is considered the most significant contributor PCOS [32]. In the female body, excessive androgens enhance the recruitment of primordial follicles into growth and development. High androgen levels also impair the selection of dominant follicles due to the premature stimulation of luteinization. Together, these pathological changes result in polycystic ovarian morphology (PCO) [7]. On a cellular level, androgens induce PCOS by initiating pathologies such as mitochondrial dysfunction, endoplasmic reticulum stress, apoptosis, and autophagy in granulosa cells and oocytes [33]. Women with PCOS have excess LH secretion [34]. LH stimulates ovarian theca cells to production androgens. In healthy women, the LH/follicle-stimulating hormone (FSH) ratio generally ranges from 1:1 to 2:1. In women with PCOS, abnormalities in the HPO axis increase LH levels relative to FSH levels. In women with PCOS, the LH/FSH ratio may be elevated to 2:1 or 3:1 [35]. Relatively low FSH levels inhibit follicular growth and maturity, impair selection of a dominant preovulatory follicle, and decrease the chance of ovulation [36]. Interestingly, some data indicate that adrenal hyperandrogenism does not exacerbate insulin resistance or dyslipidemia in women with PCOS [37].

### 2.2. PCOS and Obesity, Overweight and Other Metabolic Disturbances

Insulin resistance and compensatory hyperinsulinemia also contribute to the pathogenesis of PCOS [38]. Insulin resistance is common in PCOS patients, and insulin resistance and obesity can form a vicious cycle. Excess androgen production promotes accumulation of visceral adipose tissue, which exacerbates insulin resistance and hyperinsulinemia [39]. Likewise, obesity increases insulin resistance, exacerbating PCOS [40]. Hyperinsulinemia resulting from insulin resistance stimulates ovarian and adrenal androgen secretion and decreases hepatic SHBG synthesis, increasing free androgen levels [41]. In women with PCOS, metabolic syndrome and its individual components are prevalent, especially in women with the highest BMI and insulin levels [42]. A cross-sectional cohort study indicates that, in women with PCOS, insulin insensitivity is not present when the menstrual cycle is regular, but occurs during oligo/amenorrhoea [43]. Insulin resistance is not inextricably linked to obesity in PCOS, as it can occur in women with normal BMI diagnosed with PCOS. Excess insulin increases ovarian sensitivity to LH [7]. Further, increased blood glucose promotes peripheral insulin resistance. Hyperglycaemia affects ovarian function and could also have secondary affects by promoting the formation and accumulation of advanced glycation end products [44]. Dyslipidaemia is a common metabolic abnormality in PCOS patients. For example, LDL and TC levels are significantly higher in obese women with PCOS relative to obese women without PCOS, and in non-obese women with PCOS relative to non-obese women without PCOS [45]. Moreover, NAFLD is common in women with PCOS, and the disorders are clinically correlated [46]. In women with PCOS, circulating anti-Müllerian hormone (AMH) is higher, and is linked to antral follicle number [47]. Normocyclic women with PCOS have better metabolic parameters (BMI, HOMA-IR, and fasting insulin) compared with women with PCOS accompanied by oligo/amenorrhea [48]. Thus, women with PCOS and pathological metabolic parameters are less likely to successfully conceive.

Obesity is a chronic metabolic disease, and commonly causes comorbidities such as insulin resistance, glucose intolerance, and dyslipidemia. Over the past four decades, the number of obese women has increased from 71 million (1975) to 375 million (2014) [49]. If this trend continues, the obesity epidemic will affect 21% of women worldwide by 2025 [49]. According to WHO standards, a body mass index (BMI) greater than or equal to 25 kg/m^2^ is considered overweight, while a BMI greater than or equal to 30kg/m^2^ is considered obese [8]. Present WHO data demonstrate that in 2016, 40% of adult women were overweight and 15% were obese [9]. Adipose tissue is considered an endocrine organ that releases adipocytokines such as adiponectin, leptin, and visfatin, hormones, and growth factors, and plays a regulatory role in processes such as glucose and lipid metabolism and reproduction. Obesity is linked to female infertility through multiple complex mechanisms. In women, adipose tissue impacts the hypothalamus-pituitary–ovarian (HPO) axis. The impulses of gonadotropin-releasing hormone (GnRH) and therefore normal functioning of the reproductive (hypothalamus-pituitary-gonadal) axis is dependent upon metabolic homeostasis. In the event of energetic imbalances, reproductive function may be impaired [50]. The adipose tissue synthesizes androgens and subsequently converts androgens into estrogens [51]. Decreased sex hormone binding globulin (SHBG) levels are associated with obesity in women [52], which increases the availability of estrogens and androgens to target tissues. Central obesity is often accompanied by insulin resistance and hyperinsulinemia. The inability of insulin to suppress lipolysis in insulin-resistant adipose tissue, primarily visceral adipose tissue, increases circulating free fatty acids (FFAs). Higher circulating FFAs directly impact muscle and liver metabolism, further exacerbating insulin resistance [53]. Leptin influences steroidogenesis in the ovarian granulosa and thecal cells [54]. Compared with fertile women, infertile women have higher BMIs and levels of lipoproteins such as total cholesterol (TC), low-density lipoprotein cholesterol (LDL), and triglycerides (TGs) [55].

### 2.3. PCOS and Fertility

It is suggested that PCOS and PCOS-related morbidities are associated with altered oocyte and endometrial competence, as well as impairment of endometrial–embryonic relationship that increase the risk of infertility [56]. They can also lead to an increased risk of early and late pregnancy complications by abnormal trophoblast invasion and placentation [56]. A prospective case–control study showed that the placenta structure is altered in PCOS women with an uncomplicated pregnancy [57]. Decreased fertility potential of the PCOS affected females may be caused by independent factors, such as endometrial competence, oocyte competence/ oocyte quality (OC/OQ) and oligo- anovulatory ovarian dysfunction [58]. The high risk of OC/OQ reduction is found in the full phenotype of PCOS and many PCOS-related morbidities, such as obesity or hyperinsulinemic IR in women with PCOS [58]. Obesity, hyperinsulinemic IR, and hyperandrogenism in PCOS patients increase oxidative stress in the ovary and may affect the quality of oocytes [58]. Abnormalities and changes are observed in the endometrium in women with PCOS-related endometrial dysfunction at the cellular level [59]. It concerns: DNA synthesis and repair, cell cycling regulation and proliferation, apoptosis, glycolysis, mitochondrial metabolism, intracrine uptake and metabolism, cell transport and signaling, or the intercellular adhesion of molecules [59]. The normal functioning of endometrial receptivity is essential for embryonic implantation. In women with PCOS, many factors such as inflammation, metabolic disturbance, hormonal imbalance, and anovulation can disrupt the endometrium. This can result in endometrial hyperplasia and complications during pregnancy in the case of successful conception [60]. PCOS influences the long-term health risks in women at the reproductive and postreproductive stages of life also through metabolic effects [61]. Medications used in PCOS patients to improve endometrial function are: anti-obesity drugs, insulin-sensitizing drugs, and drug-induced endometrial shedding [59]. Insulin-sensitizing medicines such as metformin, pioglitazone, rosiglitazione, troglitazone, irisin, or inositol are used in the management of PCOS [59]. They show positive or direct metabolic effects on peripheral targets such as the endometrium [59]. The role of metformin is especially well-documented. Metfomin (1,1-dimethylbuguanide hydrochloride) is an insulin sensitizer that shows benefits in managing PCOS-related disorders, especially in overweight and obese patients [62]. Anti-obesity drugs not only reduce body weight but also may improve fertility in obese women suffering from PCOS. The following medications are used: liraglutide—receptor agonist of glucagon-like peptide-1 (GLP-1), exenatide—short acting GLP-1 analog, or orlistat—reversible inhibitor of gastric and pancreatic lipases [59]. Lifestyle change programs to reduce excessive weight are also beneficial [59]. Women suffering from PCOS have an increased risk of maternal pregnancy complications, which are: miscarriage, multiple pregnancy, gestational diabetes mellitus, pregnancy induced hypertension and pre-eclampsia or caesarean section [61]. The fetal/neonatal complications include premature delivery or SGA (small for gestational age) [61]. In patients with PCOS, the increased risk of obstetric and neonatal complications varies significantly among different phenotypes of PCOS [63]. Ovarian dysfunction and biochemical hyperandrogenism have a significant effect on this risk; however, clinical hyperandrogenism and PCO do not have a significant impact [63]. Regardless of the associations between the above factors and PCOS, no single mechanism is considered the sole causative factor of PCOS.

## 3. Cannabinoids and Cannabinoid Receptors

### 3.1. Cannabinoid Synthesis and Classification

Cannabinoids are divided into three groups: endocannabinoids (eCBs) and their metabolites, phytocannabinoids, and synthetic cannabinoids.

#### 3.1.1. Endocannabinoids

Endocannabinoids include two primary compounds, anandamide (AEA) [64] and 2-arachidonoylglycerol (2-AG) [65], together with other endogenous cannabimimetic molecules and endocannabinoid-like compounds. Other endogenous cannabimimetic molecules include oleamide (ODA), virodhamine (O-AEA), noladin ether (2-AGE), N-arachidonoyldopamine (NADA), and N-arachidonoylglycine (NAGly) [66]. The main endocannabinoid-like compounds include oleoylethanolamide (OEA), palmitoylethanolamide (PEA), stearoylethanolamide (SEA), and linoleoylethanolamide (LEA) [67]. The primary receptors for both major endocannabinoids are cannabinoid receptor type 1 (CB1R) and CB2R (Table 2).

AEA is the best-described eCB, belonging to the N-acylethanolamines (NAE). AEA synthesis is initiated by increased intracellular calcium levels, which activate N-acetyltransferase (NAT), which catalyzes the transfer of arachidonic acid (AA) from the Sn-1 position of phosphatidyl choline to phosphatidylethanolamine, forming N-arachidonoyl phosphatidylethanolamine (NAPE) [68]. Intracellular NAPE concentrations are low, as NAPE is hydrolyzed by NAPE-specific phospholipase D (NAPE-PLD) to AEA [69,70]. AEA can also be synthesized in the presence of phospholipase C, A2, or α/β-hydrolase 4 (Abh4) [68,71]. AEA is also hydrolyzed into free AA and ethanolamine, which is catalyzed by fatty acid amide hydrolase (FAAH) [72]. AEA is the derivative of unsaturated fatty acids and is well-known to be metabolized by cyclooxygenase (COX-2), lipoxygenase (LOX), and cytochrome P450 [73]. These reactions form prostanoids, some of which are PPAR ligands [74].

The other primary eCB is 2-AG. 2-AG belongs to the monoacylglycerol (MAG) class of compounds. Two metabolic pathways can synthesize 2-AG. However, Sn-2 arachidonic acid-containing glycerophospholipids from the plasma membrane are the primary substrates of all 2-AG-synthesizing pathways [75]. The primary precursors are inositol phospholipids with a 2-arachidonoyl group, which are hydrolyzed by phospholipase C to form 2-arachidonoyl-diacylglycerol (a DAG). Next, a specific diacylglycerol lipase (DAGL) deacylates mentioned diacylglycerol (DAG) to form 2-AG. 2-AG can also be hydrolyzed from other glycerophospholipids such as phosphatidic acid and phosphatidylcholine [76,77,78]. Two human DAGL isoforms have been identified, DAGLα and DAGLβ [79]. The primary synthetic enzyme for 2-AG in the central nervous system (CNS) is DAGLα. 2-AG is generally hydrolyzed to AA and glycerol by enzymes such as monoacylglycerol lipase (MAGL), FAAH, α/β-hydrolase domain-containing (ABHD) 6, and ABHD12. In other pathways, 2-AG is metabolized by cyclooxygenase-2 (COX-2) or lipoxygenases [80].

AEA and 2-AG play roles in multiple biological processes in the CNS and peripheral nervous system (PNS) [81]. AEA is a partial agonist of CBRs, while 2-AG is a full agonist of CBRs [82].

#### 3.1.2. Plant Cannabinoids

Phytocannabinoids are natural CBs found in the cannabis plant. The major species of plant cultivated for use as marijuana is cannabis sativa, with subspecies such as cannabis indica and distinct strains within these subspecies [83]. Marijuana (also referred to as cannabis) is a psychoactive drug derived from dried flowers of the cannabis plant.

There are nearly 150 different CBs found in cannabis plants [84]. The primary CBs are Δ9-tetrahydrocannabinol (Δ9-THC) and cannabidiol (CBD). Other additional cannabinoids, referred to as minor cannabinoids, include Δ9-tetrahydrocannabinolic acid (Δ9-THCA), Δ(9)-tetrahydrocannabivarin (Δ9-THCV), cannabigerol (CBG), cannabichromene (CBC), cannabinol (CBN), and cannabidivarin (CBDV) [84]. Δ9-THC is the primary psychoactive cannabinoid in marijuana [85]. Cannabis is the most widely used illicit drug worldwide [86]. Δ9-THC binds primarily to CB1R but also weakly binds CB2R. Δ9-THC binds primarily to CB1R, but also weakly binds CB2R [85]. CBD does not have psychomimetic effects, but has analgesic and anti-inflammatory properties [87].

#### 3.1.3. Synthetic Cannabinoids

Synthetic CBs are manmade molecules that bind the same receptors as natural CBs. Many synthetic CBs have been produced, including WIN 55212-2, CP55940, AM-2201, HU-210, and JWH-018 [88].

Synthetic Δ9-THC variants have been developed as research tools to better understand the physiological ECS, or as potential therapeutics.

### 3.2. Structure and Function of Cannabinoid Receptors

#### 3.2.1. Plasma Membrane Receptors

The human *CNR1* gene, which encodes CB1R, is located on human chromosome long arm 6q14-15 [89]. Several transcript variants coding for two different protein isoforms have been described [90]. For example, in human hepatocytes, *CB1R* mRNA consists of four exons. Exon 1 contains two splicing sites (1A and 1B), while exon 4 contains four (4A–D) [91]. Due to the alternative splicing phenomenon, six transcript variants can be produced. The translation of intact exon 4 produces CB1 full amino acid length receptor [91]. Isoforms of that receptor are expressed differently in various human tissues. The human *CNR2* gene is located on a short arm of chromosome 1p36.11 [92]. Three transcript variants, the X1, X2, and X3 isoforms, translate into three splice variants of CB2R [93].

**Figure 1:**
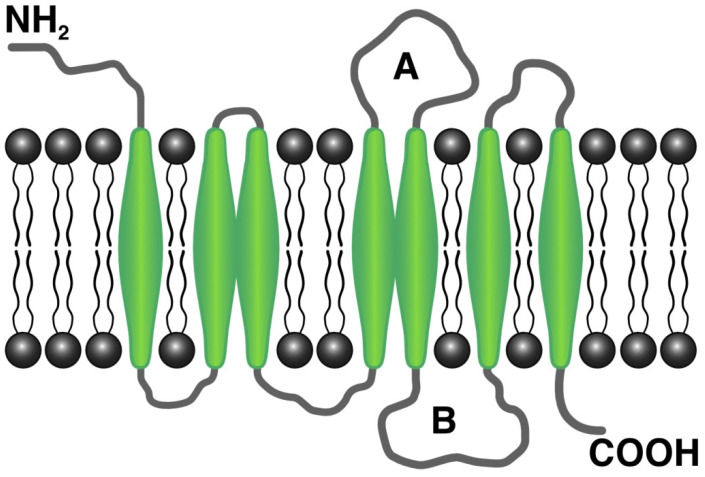
CB1R structure. (A) Second outer loop agonist attachment site. (B) Third inner loop G protein attachment site.

The cannabinoid receptors CB1R and CB2R both belong to the class A family of G protein-coupled receptors (GPCRs) [94]. GPCRs comprise a single polypeptide chain that spans the cell membrane seven times with the N terminus protruding extracellularly and the C terminus located in the cytoplasm. Receptor binding activates an intracellular signaling cascade. CB1R is comprised of 472 amino acids (molecular mass 64 kDa) [90] and contains seven transmembrane domains, three extracellular domains, and three intracellular domains [90]. Figure 1 shows the structure of CB1R. CB2R comprises 360 amino acids, and its structure and function resemble that of CB1R [93]. Both CB1 and CB2 receptors transmit signals to intracellular machinery via Gi and Go proteins [95]. In specific conditions and under the influence of some agonists, signal transmission is activated by Gs or Gq/11 proteins [82]. Agonist binding to cannabinoid receptors results in G protein decomposition into three subunits, α, β, and γ. The α subunit inhibits intracellular adenylyl cyclase, decreasing cAMP concentration. cAMP decrease causes a blockade of calcium channels, the activation of potassium channels, and decreased neurotransmitter secretion. For example, the activation of CB1R in neuron synapses inhibits the release of neurotransmitters such as acetylcholine, noradrenaline, dopamine, serotonin, glutamate, and gamma-aminobutyric acid (GABA) [96]. The β and γ subunits, in addition to Gi/Go proteins, trigger the Mitogen-activated protein kinase (MAPK) cascade. MAPK signaling affects cell metabolism, growth, migration, differentiation, and apoptosis [97]. The activation of CB1R also influences the activities of protein kinase A and C.

Cannabinoid ligands bind to different sensor proteins such as transient receptor potential channels (TRPs), especially transient receptor potential cation channel subfamily V member 1 (TRPV1), GPR18, GPR55, GPR119, glycine receptors, serotonin receptors (5-HT), opioid receptors, and PPARs [98,99].

#### 3.2.2. Intracellular Receptors

CB1R is primarily localized to the plasma membrane, but some studies have reported that CB1R is localized to intracellular membranes in some cell types. CB1R can localize to several organelles, including mitochondria (mtCB1R—mitochondrial CB1R), lysosomes/acid-containing endosomes that do not contribute to cell surface re-population [100,101], and endosomes, in which plasma membrane CB1R is internalized [102]. The mitochondrial localization of CB1R has been detected in murine skeletal muscle fibers [103], brain neurons and astrocytes [104,105], and ovarian steroidogenic cells [106]. In the reproductive system, CB1R is present in in situ ovarian interstitial glands and the mitochondrial membranes of progesterone-producing cells [106]. Endocannabinoids could affect progesterone synthesis in these cells via CB1R, because progesterone synthesis is processed in mitochondria [106]. Experimental mice studies that use immunoelectron microscopy and reverse transcription polymerase chain reaction (RT-PCR) methods revealed that mtCB1R is present in the mitochondria of striated gastrocnemius and rectus abdominis skeletal muscles and myocardial muscles [103]. Δ9-THC activation of mtCB1R in myocardial cells decreases mitochondrial respiration [103]. In striated muscle, mtCB1R activation contributes to the regulation of mitochondrial oxidative activity, most likely via enzymes involved in metabolism of pyruvate, a major substrate for synthesis of acetyl-CoA, which is shunted into the Krebs cycle [103]. In mice, mtCB1R is also present in neurons, where mtCB1R directly controls neuronal respiration and ATP production [104]. The activation of mtCB1R in neuronal mitochondria decreases cyclic AMP (cAMP) concentration, protein kinase A activity, complex I enzymatic activity, and respiration [104]. The activation of mtCB1R in astroglia disrupts glucose metabolism and lactate production in the mouse brain [107].

### 3.3. Physiological Roles of Cannabinoids

#### 3.3.1. Role of CBs in Carbohydrate Metabolism, Lipid Metabolism, and Obesity

Cannabinoid receptors are expressed in nearly every tissue of the human body. CB1R expression has been detected in many regions of the human CNS [108]. In the hypothalamus, CB1R is expressed in the ventromedial nucleus of the hypothalamus and in the paraventricular nucleus [109]. The effect of CB1R activation in mesolimbic (dopaminergic) and hypothalamic neurons on food intake and in energetic homeostasis has been described [110]. Fasting activates CB1R to induce food intake by regulating levels of appetite-stimulating factors [111]. Hunger is generally provoked by hormonal changes such as ghrelin increase and leptin decrease, but the eCBs 2-AG and AEA bind hypothalamic CB1R to trigger the hunger response [112]. Direct activation of CB1R by AEA stimulates food intake [113]. Clinical trials by Foltin et al. demonstrated that low-dose marijuana does not affect food intake, but that higher doses increase daily caloric intake due to increased food consumption between meals rather than increased meal size [114]. A cross-sectional study has demonstrated that excessive cannabis use is associated with decreased incidence of obesity compared to non-users [115]. This study is a premise for this idea because it is understood that marijuana smoke has a lot of chemical substances in it. Furthermore, anandamide (AEA) in the nucleus accumbens intensifies the reward response to sweet flavors [116]. Δ9-THC also amplifies dopamine release in the nucleus accumbens shell and intensifies the taste reaction [117]. CB1R is expressed in the brain at much higher levels than CB2R [118], but CB2R also has a regulatory effect on food intake. CB2R stimulation decreases food intake and weight gain without negatively impacting mood and could thus alleviate obesity [119]. Dysfunction of the ECS could contribute to obesity. In ob/ob mice with deficient leptin synthesis and resultant impairment of food satiety and development of obesity, eCBs levels are increased in the hypothalamus [120]. In rodent models, short-term fasting increases hypothalamic eCBs levels relative to animals fed ad libitum [120]. These findings suggest that CB1R antagonism in the CNS could suppress appetite. The selective CB1R antagonist SR141716A, which has low or no affinity to other receptors in vitro, is commercially known as Rimonabant [121], and freely penetrates the brain–blood barrier [122]. Sanofi Aventis conducted research evaluating Rimonabant as an anti-obesity therapy that suppresses appetite. Animal tests demonstrated decreased food intake and subsequent decreased body mass in animals treated with Rimonabant [123,124,125,126,127], which was recapitulated in humans. Patients treated with Rimonabant had decreased BMI relative to placebo-treated patients [26]. Rimonabant (SR141716A) was introduced in the European Union under the brand name Accomplia in 2006 as an intervention for weight reduction [128]. However, Accomplia was withdrawn from the market in 2008 due to side effects such as depression and anxiety [128].

The PNS also regulates energetic homeostasis by coordinating interactions between the gastrointestinal tract (GI), pancreas, adipose tissue, and muscles. Elements of the ECS, such as CB1R, are present in human peripheral organs involved in metabolic homeostasis, including white adipose tissue [129], skeletal muscle [130], pancreas [131], gut [132], and liver [133]. As previously mentioned, CBs are present in different tissues related with obesity. CB1R is expressed in the adipose tissue in both humans [129] and rodents [134]. CB1R expression patterns fluctuate during adipose tissue differentiation. CB1R expression is higher in mature fat cells than in pre-adipocytes [135]. Interestingly, mature adipocytes from both visceral and subcutaneous fat tissue express both CB1R and CB2R [135]. The influence of CB1R agonists, especially AEA and 2-AG, on adipogenesis and lipogenesis has been reported. Activation of these receptors by AEA and 2-AG in white adipose tissue in vitro prompts fatty acids (FA) synthesis, TG accumulation, and decreased lipolysis [112].

CB1 receptors are involved in the regulation of insulin resistance. In human islets, CB1R is highly expressed in α-cells (glucagon-secreting) and modestly expressed in β-cells (insulin-secreting) [131]. However, CB2R is highly expressed in δ-cells (somatostatin secreting) but is not expressed in α- or β-cells [131]. The ECS regulates insulin levels, glucose uptake, and glucose utilization, impacting glucose tolerance. The stimulation of CB2R in murine pancreatic islets decreases insulin secretion [136]. CB1R plays important roles in the GI tract. CB1R activation by eCBs in the GI tract induces GI motility, increases vasodilation, and decreases secretion of acid and fluid, influencing nutrient absorption [137,138]. CBs also affect lipid metabolism, insulin sensitivity, and the development of hepatic steatosis via CB1R [139]. In murine hepatocytes, CB1R activation stimulates the expression of lipogenic enzymes such as fatty acid synthase and increases de novo fatty acid synthesis, leading to lipid accumulation and, ultimately, steatosis [140]. In murine hepatocytes, feeding a high-fat diet increases AEA level, CB1R density, and basal rates of fatty acid synthesis, which is impaired by CB1R blockade [140]. A cross-sectional, population-based study demonstrated that chronic cannabis use potentially decreases hepatic steatosis, decreasing the prevalence of NAFLD in cannabis users compared with non-users [141].

#### 3.3.2. Role of CBs in Embryo Implantation and Female Fertility

A prospective cohort study of women and a cross-sectional study of men have demonstrated that cannabis, specifically Δ9-THC, impairs both female and male fertility [142,143]. Elements of the endocannabinoid pathways are present in the female reproductive system, including the uterine endometrium [18] and myometrium [19], the oviducts [17], and the ovaries [16]. In normal human ovaries, CB2R expression is higher than CB1R expression in ovarian cells [16]. Hormones influence AEA production in the ovaries, which affects ovarian processes such as folliculogenesis, preovulatory follicle maturation, oocyte maturity, and ovulation [16]. Further, the expression of CB1R and CB2R and endocannabinoid-metabolizing enzymes such as NAPE-PLD and FAAH fluctuate with the menstruation cycle in the endometrium [18]. In premenopausal women, circulating AEA fluctuates during the ovulatory cycle, with the highest level during ovulation and the lowest level in the late luteal phase [144]. Peak circulating AEA levels correlate with serum levels of gonadotropin (FSH, LH) and estradiol (E2), but not with serum progesterone (P4) [144]. Progesterone and estrogen downregulate FAAH activity and expression in the murine endometrium in early pregnancy [145], potentially allowing the accumulation of anandamide, which could play an important role in altering the endometrium during pregnancy [145].

The endocannabinoid pathway is involved in the processes of insemination, ovum transport in the oviduct, early stages of embryonic development, and embryonic implantation in the uterus [20,21,22]. The involvement of CBRs and their agonists in early embryonic development and implantation are crucial. AEA, PEA, and OEA are present in seminal plasma, mid-cycle oviductal fluid, follicular fluid, and amniotic fluid or milk [146]. Experimental in vitro (blastocyst growth and hatching) and in vivo (blastocyst implantation) research in mice has demonstrated that changing uterine AEA levels are related to uterine receptivity to embryonic implantation [147]. In human immunohistochemical studies of women with ovarian stimulation undergoing in vitro fertilization (IVF) and intracytoplasmic sperm injection (ICSI) with embryo transfer (ET) in the retrieved oocytes, mean follicular fluid AEA concentrations was higher in follicles with mature oocytes than in follicles with immature oocytes [16]. This suggests the potential involvement of AEA in oocyte maturation. RT-PCR research in mice identified the expression of both CB1R and CB2R in preimplantation embryos [148]. CB2R mRNA is detectable at the single-cell blastocyst stage, while CB1R mRNA is detectable at the four-cell blastocyst stage [148]. Uterine AEA levels at different phases of receptivity and blastocyst CBR expression are correlated in mice [149]. Specifically, decreased AEA binding to the blastocyst is important for the onset of implantation [149]. In in vitro studies, synthetic (WIN 55,212-2, CP 55,940) and natural cannabinoid (Δ9-THC, AEA) agonists arrested the development of two-cell mice embryos in a dose-dependent manner, primarily between the four-cell and eight-cell stages [148]. Interestingly, neither CBD nor AA had this effect [148]. The in vitro arrest of embryo development by AEA and 2-AG was reversed by CB1R antagonists (SR 141716A- Rimonabant, and AM 251), but not a CB2R-selective antagonist (SR144528) [150]. Moreover, the selective CB2R agonist AM 663 did not affect embryo development [150]. This underscores the importance of CB1R in embryonic development. Research that uses immunostaining method in knockout mice demonstrated that in Cb1r-/-, Cb2r-/-, and Cb1r-/-/Cb2r-/- embryos on the third day of pregnancy (in the oviduct) and on the fourth day of pregnancy (in the uterus) undergo asynchronous development in comparison to wild-type embryos [149].

Fertilization occurs in the ampulla of the fallopian tube. The embryo is then transported to the uterus, where it is implanted. Transportation involves the cannabinoid system. Dysfunctional embryo transport along the oviduct can cause ectopic pregnancies due to embryo attachment to the wall of fallopian tube. In mice, CB1R, but not CB2R, is expressed in the oviducts on days 1–4 of pregnancy [151]. In Cb1r-/- and Cb1r-/-/Cb2r-/- mice, embryo detention in the oviduct was increased relative to Cb2r-/ mice or wild-type [151]. This suggests that the oviduct is a target for endocannabinoid signaling, which influences embryo transport in the fallopian tubes [151]. Subsequently, the embryo reaches the uterus and implants in the uterine wall. This process is also regulated by hormones and CBs. In mice, AEA level in the peri-implantation uterus changes with the state of pregnancy [147]. AEA concentrations are lowest at the site of embryo implantation and highest in the interimplantation sites and the pseudopregnant uterus during the nonreceptive phase [147]. Cohort study of women, using high-performance liquid chromatography-mass spectrometry (HPLC-MS) has demonstrated that plasma AEA levels change during the menstrual cycle and pregnancy [152]. In the luteal phase, circulating AEA is lower than in the follicular phase [152]. Importantly, the follicular phase is the implantation window period. In pregnant women, circulating AEA levels are higher in the first trimester than in the second and third trimesters [152]. This suggests that low circulating AEA levels are needed for successful embryo implantation and pregnancy progression [152]. Interestingly, in mice, an inverse relationship is present between the NAPE-PLD enzyme, which is involved in AEA synthesis, and the FAAH enzyme, which is involved in AEA degradation, at uterine implantation sites, and at interimplantation sites [153]. NAPE-PLD accumulation is higher in the endometrium resistant to implantation than in the receptive site of the endometrium [153]. FAAH is lower in the interimplantation region of the endometrium than in the implantation site [153]. Moreover, the implanting blastocyst also highly expresses FAAH [153].

#### 3.3.3. Role of CBs in PCOS

Above, we describe PCOS and the cannabinoid system with special regard to its role in metabolic homeostasis and the female reproductive system. In this section, we will focus on data demonstrating a significant relationship between eCBs and PCOS. In an immunohistochemical study of endometrial biopsies from women with and without PCOS, CB1R levels did not differ between groups or fluctuate with the menstrual cycle [154]. However, endometrial FAAH levels were lower in women with PCOS than in control subjects, and FAAH levels were significantly elevated in the secretory phase compared with the menstrual and proliferative phases [154]. Case–control human study with use RT-PCR method demonstrated that CB1R and CB2R mRNA expression and level of AEA and 2-AG (HPLC-MS) were significantly higher in peripheral blood mononuclear cells (PBMCs) from women with PCOS than in those of women without PCOS [155]. In adipose tissue, the expression of CB1R, but not CB2R, is significantly higher in women with PCOS [155]. A study using immunohistochemical method and liquid chromatography–electrosprayionization–mass spectrometry (LC-ESI-MS) compared women with PCOS and infertile women without PCOS, as well as women with PCOS before and after treatment with Diane-35 (ethinyl estradiol plus cyproterone acetate) and metformin [156]. Circulating AEA was higher in women with PCOS relative to infertile women without PCOS [156]. The endometrial expression of FAAH was lower in women with PCOS relative to infertile women without PCOS, but no significant difference in endometrial cannabinoid receptor expression was detected [156]. Endometrial FAAH expression increased after treatment with Diane-35 and metformin in women with PCOS [156]. Circulating 2-AG levels were similar in women with PCOS and healthy controls [157]. However, women with phenotype A exhibited significantly lower endocannabinoid levels than women with phenotype B or healthy women [157]. A cohort study of AEA levels in women with revealed interesting findings. There were no differences in AEA levels between healthy women and women with PCOS. However, among women with PCOS, a significant difference in AEA levels was detected between body types: AEA levels were significantly higher in the gynoid-type group relative to the android-type group [158]. CB1R are found in the human hypothalamic–pituitary–adrenal (HPA) axis, as well as in the hypothalamus. CB1R is expressed in the pituitary gland [159] and adrenal cortex [160]. At the adrenal level, the ECS could directly inhibit adrenocortical steroidogenesis (corticosterone and aldosterone) via CB1Rs [160].

In women with PCOS, mitochondrial dysfunction occurs in granulosa cells, and is accompanied by abnormal glycolysis, which affects the switch from metabolic to glycolytic metabolism [161]. Moreover, mitochondria produce most of the reactive oxygen species (ROS) that cause oxidative stress, and is also associated with IR. Consequently, mitochondrial dysfunction potentially has a central role in the pathogenesis of PCOS [162]. Accordingly, mtCB1R is implicated in cell metabolism. For example, in mouse neurons, mtCB1R directly controls respiration and ATP production [104].

## 4. PPARs

### 4.1. PPARs and Their Ligands

**Figure 2:**
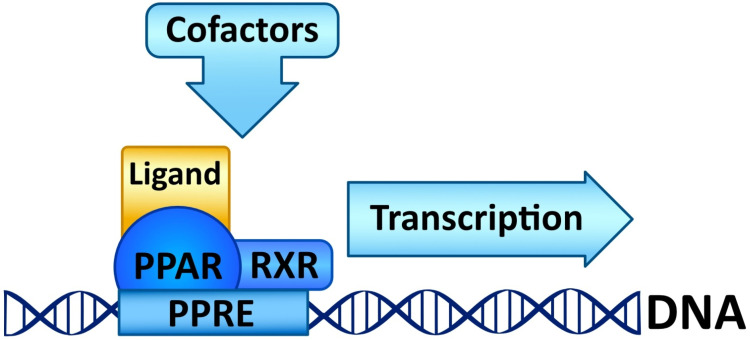
Activation of peroxisome proliferator activated receptors.

Peroxisome proliferator-activated receptors (PPARs) are members of large nuclear receptor superfamily of transcription factors, which regulate the expression of specific genes. NRs include thyroid hormone receptor (THR), steroid hormones receptors, vitamin D3 receptor (VDR), retinoic acid receptor (RAR), and PPARs. PPARs regulate expression of genes related to metabolic homeostasis, glucose and lipid metabolism, adipogenesis, and inflammation [163]. Their function as transcription factors depends on the type of ligand. Gene transcription is initiated by PPAR ligand binding. Next, PPARs heterodimerize with the retinoid X receptor (RXR), another ligand-activated nuclear receptor. The PPAR–RXR heterodimer binds to specific response elements in the promoters of target genes, termed peroxisome proliferator hormone response elements (PPREs) [164]. Cofactor proteins such as coactivators or corepressors may modulate the transcriptional activity of the PPAR–RXR heterodimer via binding [165]. The activation of PPARs is shown in Figure 2.

Three types of PPARs have been identified in mammals: α, β/δ, and γ [166]. These three PPAR isotypes are also referred to as NR1C1 (α), NR1C2 (β/δ), and NR1C3 (γ) [167,168,169].

PPARs are comprised of six functional domains, A–F. PPAR protein architecture consists of N-terminal A/B domains responsible for transcriptional activation, also referred to as activation function-1 (AF-1), the C domain responsible for DNA recognition and protein–protein interactions, also referred to as the DNA-binding domain (DBD), the flexible hinge D domain, the ligand-binding E/F domains in the C-terminus, also referred to as the ligand-binding domain (LBD), and the AF-2 domain [170].

The gene transcription process is identical in all three types of PPARs [171]. PPAR types vary in ligand specificities, biological activities, and tissue locations [25]. Endogenous PPAR ligands include lipids such as free fatty acids FFAs and eicosanoids [172].

#### 4.1.1. PPARα

PPARα is expressed in multiple tissues, but is most highly expressed in the liver, kidney, heart, skeletal muscle, and small intestine [167]. The human *PPARα* gene is located on the long arm of chromosome 22 (22q12-q13.1) and consists of eight exons [173]. PPARα receptors control expression of genes involved in fatty acid metabolism [174].

PPARα ligands are fatty acid derivatives from lipid catabolism, lipolysis, and lipogenesis [172]. Saturated FA are weak PPARα ligands compared with unsaturated fatty acids [175]. Thus, the endogenous PPARα ligands are specific unsaturated FA, specific phospholipids, leukotriene B4 (LTB4), and 8(S)-hydroxyeicosatetraenoicacid (8S-HETE) [176]. The most common synthetic PPARα ligands are fibrates such as clofibrate, fenofibrate, bezafibrate, gemfibrozil [176]. Fibrates are used to treat dyslipidemia, primarily hypertriglyceridemia, and hypercholesterolemia [177].

#### 4.1.2. PPARβ/δ

PPARβ/δ is expressed in many tissues. It is most robustly expressed in the placenta and thyroid, but is also expressed in tissues with high lipid metabolism, such as the liver, adipose tissue, skeletal muscle, and small intestine [168]. The human *PPARβ/δ* gene is composed of nine exons and is encoded on the short arm of the chromosome 6 (21.2–21.1) [178]. PPARβ/δ controls fatty acid catabolism in skeletal muscle and brown adipose tissue, contributing to systemic lipid metabolism [166,168]. The activation of muscle cell PPARβ/δ induces energy production via fatty acid oxidation rather than glycolysis [179]. This metabolic shift can increase muscle endurance by preserving glucose [179].

Endogenous PPARβ/δ ligands are polyunsaturated fatty acids (PUFAs) and their metabolites, such as linoleic acid, arachidonic acid, prostacyclin (prostaglandin I2, PGI2), 15S-hydroxyeicosatetraenoic acid (15-HETE), and 13-hydroxyoctadecadienoic acid (13-HODE). The most commonly used synthetic PPARβ/δ agonists are GW0742 and GW501516 [180].

#### 4.1.3. PPARγ

The PPARγ receptor is expressed in diverse tissue types but is most robustly expressed in adipocytes [169]. The *PPARγ* gene is located on the short arm of chromosome 3 (3p25) and is composed of nine exons [181]. Three mRNA splice variants, *PPAR-γ1*, *PPAR-γ2*, and *PPAR-γ3* are generated [169]. Two PPARγ protein isoforms, PPARγ1 and PPARγ2, are translated from these splice variants. The *PPARγ1* and *PPARγ3* mRNA splice variants both translate to the PPARγ1 isoform, while the *PPARγ2* mRNA splice variant translates to the PPARγ2 isoform, which contains an additional NH2-terminal region consisting of 30 amino acid residues [169]. Both PPARγ1 and PPARγ2 play critical roles in adipocyte differentiation and glucose metabolism but differ in expression pattern. PPARγ1 is shorter and is expressed in nearly all brain and immune cells, while the PPARγ2 receptor is expressed primarily in white and brown adipose tissue [170].

Endogenous PPARγ receptor ligands include unsaturated FA such as arachidonic acid, phospholipids, lysophosphatidic acid, and linoleic acid [175,182], and products of the cyclooxygenase and lipoxygenase pathways such as 9-hydroxyloctadecadienoic acid (9-HODE), 13-hydroxyoctadecadeienoic acid (13-HODE), 15-hydroxyeicosatetraenoic acid (15-HETE), and 15-deoxy-D12,14-prostaglandin J2 (15d-PGJ 2) [176]. Thiazolidinediones (TZDs), also known as glitazones, are the most commonly used synthetic PPAR*γ* ligands, including pioglitazone, rosiglitazone, troglitazone, and ciglitazone [183]. Thiazolidinediones are full PPARγ agonists and are used as insulin sensitizers [183]. Glitazones are used to treat diabetes mellitus.

### 4.2. Physiological Role of PPARs

#### 4.2.1. Role of PPARs in Energy Metabolism and Obesity

Fibrates are class of compounds that activate PPARα. Fibrates increase the oxidation of FA in the liver and muscles and decrease liver lipogenesis, thus reducing the secretion of very-low-density lipoprotein (VLDL) and triglycerides [184]. Fibrates are effective in decreasing serum TGs and increasing serum HDL, and in some patients, also decrease serum levels of LDL, TC, and apolipoprotein B (Apo-B). Fibrates improve plasma HDL levels by upregulating hepatic production of Apo-AI and Apo-AII [177]. Different fibrates have similar effects on dyslipidemia, but subtly differ in their impact on glucose metabolism, insulin resistance, intermittent claudication, and effects on microvascular complications of diabetes mellitus [177,184]. Bezafibrate, unlike other fibrates, is a pan-PPAR (α, β/δ, γ) agonist [185]. Its effects on glucose metabolism and insulin resistance differ from that of other fibrates. Bezafibrate stabilizes insulin sensitivity and pancreatic β-cell function, decreases blood glucose and HbA1C, and increases serum adiponectin [186,187,188,189,190]. PPARα plays an important role in hepatic lipoprotein metabolism. Sun et al. demonstrated that Hepatic Krüppel-like factor 16 (KLF16) promotes hepatic lipid catabolism and insulin sensitivity via PPARα [191]. Hepatic steatosis and insulin resistance are increased by deficient fatty acid oxidation, which leads to lipid accumulation [191].

PPARβ/δ influences glucose homeostasis and fatty acid oxidation. PPARβ/δ activation induces expression of genes related to fatty acid oxidation and energy dissipation in adipose tissue in mice [192]. This improves the lipid profile and decreases adiposity in *ob/ob* mice and wild-type mice fed a high-fat diet [192]. Consistent with this finding, *Pparβ/δ^-/-^* mice are prone to diet-induced obesity [192]. This suggests a role for PPARβ/δ as a regulator of adipose tissue fatty acid oxidation [192]. PPARβ/δ is also highly expressed in rat pancreatic islets and in insulinoma cells (cell line INS-1E) [193]. Pancreatic β-cell functioning is dependent on proper maintenance of mitochondrial metabolism, and Ravnskjaer et al. suggested that the activation of PPARβ/δ and RXR prevents pancreatic β-cell dysfunction [193]. The only source of insulin in human body is β-cells in pancreatic islets, and mitochondrial function is imperative for glucose-stimulated insulin secretion. Interestingly, experimental studies of mice with intestinal epithelial cell-specific deletion of *Pparβ/δ* indicated that intestinal PPARβ/δ is protective against diet-induced obesity, insulin resistance, and dyslipidemia [194]. GW501516, also known as GW-1516, cardarine, or endurobol, was considered as an intervention for dyslipemia [195]. However, despite initial promising findings that GW501516 alleviates dyslipidaemia, further animal studies revealed tumorigenic effects in organs such as the liver, stomach, tongue, skin, bladder, ovaries, womb, and testes, precluding its therapeutic use [196]. Endurobol can be illegally abused by athletes for performance-enhancing purposes [195].

PPARγ regulates fatty acid synthesis and storage in adipose tissues, and affects glucose metabolism. PPARγ activation initiates transformation of preadipocytes into mature adipocytes [197]. Importantly, this process occurs in subcutaneous but not omental adipose tissue [197]. A study of obese Zucker rats demonstrated that troglitazone did not change the total weight of white adipose tissue, but did increase the number of small adipocytes (morphometry analysis) in the retroperitoneal and subcutaneous adipose tissues [198]. The PPARγ2 subtype plays a significant role in adipocyte differentiation [199]. Glitazones (thiazolidinediones) are used to treat DM-2 due to their therapeutic effects on glycemic control and insulin resistance. Two thiazolidinediones, rosiglitazone and pioglitazone, are used in clinical practice for the management of DM-2. Thiazolidinediones improve the response to insulin, increasing insulin sensitivity in crucial tissues, subsequently promoting insulin-dependent glucose absorption in muscle and fat, increased adiponectin levels (a cytokine secreted by adipose tissue that increases insulin sensitivity and fatty acid oxidation), and decreased hepatic gluconeogenesis [200,201,202,203,204]. PPARγ activation by glitazones alters expression levels of many genes involved in glucose and fatty acid metabolism, such as lipoprotein lipase, glucokinase, and fatty acyl-CoA synthase [205]. The influence of PPARγ target genes on the improvement of IR is due to upregulation of adiponectin and GLUT4 expression and suppression of tumor necrosis factor-α (TNF-α) signaling in adipocytes. Increased insulin-sensitive glucose uptake in adipocytes and skeletal muscle is due to higher GLUT 4 expression [206]. However, increased body weight is a well-known side effect of glitazones. Interestingly, TZDs decrease the mass and activity of visceral adipose tissue, but increase the mass of subcutaneous adipose tissue [207]. A double-blind randomized trial of patients suffering from DM-2 compared the effects of pioglitazone and metformin [208], revealing that pioglitazone decreases TG levels and increases HDL levels more significantly than metformin [208]. However, LDL and TC levels were higher in patients treated with pioglitazone than in patients treated with metformin [208].

#### 4.2.2. Role of PPARs in Embryo Implantation and Female Fertility

PPARs play roles in the male and female reproductive tracts and influence fertility. PPARs and RXR are expressed in tissues of the reproductive system, including the testicles, ovaries, uterus, hypothalamus, pituitary gland, and in early embryos and developing fetuses [209]. All three PPAR isotypes are expressed in the uterine and ovarian tissue. In the rat ovary, *Pparα* mRNA is primarily detected in the theca and stroma cells, and *Pparβ/δ* mRNA is expressed in the whole ovary [210]. Expression of *Pparγ* mRNA is restricted primarily to granulosa cells in developing follicles during pseudopregnancy and the estrous cycle [210]. PPARγ is expressed at the beginning of folliculogenesis during the primary/secondary follicle stages, is further upregulated leading up to the large follicle stage, and is downregulated following the LH surge [211,212,213]. In sheep, endometrial PPARβ/δ expression is consistent, while PPARα and PPARγ expression levels are regulated during the peri-implantation period [214]. PPARs affect physiological processes in trophoblasts such as differentiation, maturation, proliferation, migration, fusion, invasion, and secretion by regulating glucose metabolism, lipid metabolism, and amino acid metabolism [215]. In the syncytiotrophoblast layer of the human placenta, PPARα, PPARβ/δ, and PPARγ are present [216]. The absence of PPARγ in mouse embryos (*Pparγ^-/-^*) results in embryo lethality due to placenta alteration and malformation of the vascular labyrinth [217]. In vitro studies using expression analysis methods (immunohistochemical staining, RT-PCR) and transcriptional analysis methods (electrophoretic mobility shift assay (EMSA) and luciferase assays) of human cytotrophoblast and syncytiotrophoblast cells demonstrated that the activation of PPARγ by a specific agonist (rosiglitazone) is associated with increased hCG secretion and transcription levels of hCG gene [218]. Pioglitazone improves insulin sensitivity by modifying insulin-like growth factor-I (IGF) signaling, promoting extravillous trophoblast cell migration [219]. Pioglitazone, a PPARγ agonist, significantly upregulates visfatine expression in BeWo cells, which counteracts the inhibitory effect of IL-6 [220]. It is suggested that TZDs such as pioglitazone can promote energetic metabolism of trophoblastic cells by upregulating visfatin, maintaining the function of the placenta, and improving pregnancy outcome [220]. Interestingly, in humans, maternal circulating adiponectin activates placental PPARα in primary trophoblast cells [221]. In rats, elevated uterine PPARβ/δ expression in the implantation sites and decidual cells suggests a significant role for PPARβ/δ during implantation and decidualization [222]. In mice, fenofibrate inhibits estrogen synthesis in the ovary by suppressing mRNA expression, and PPARα is indispensable for this inhibitory effect [223]. The activation of PPARβ/δ by PGI2 accelerates blastocyst hatching in mice [224].

#### 4.2.3. Role of PPARs in PCOS

PCOS is associated with abnormalities in the reproductive tract, especially ovarian function and metabolic homeostasis. Above, we discussed the role of PPARs in the metabolic system in the context of female reproduction, and in this subchapter, we will focus on the connection between PPARs and PCOS. Thiazolidinedione is used as a therapeutic intervention for women with PCOS. In porcine ovarian follicles, rosiglitazone upregulates PPARγ expression and progesterone secretion, decreases androstenedione and testosterone secretion, and does not change estradiol secretion [225]. Interestingly troglitazone inhibits insulin and LH co-stimulated de novo androgen biosynthesis in porcine thecal cells in vitro [226]. A meta-analysis designed to assess the role of TZDs in PCOS demonstrated that TZDs effectively decrease insulin and fasting blood glucose levels in patients with PCOS [227]. However, TZDs do not effectively decrease androgen levels, and can increase body weight [227]. Thiazolidinediones and metformin/thiazolidinedione co-treatment ameliorate dyslipidaemia more effectively than metformin alone in women with PCOS [228]. A randomized controlled trial study investigated the metabolic and ovarian effects of rosiglitazone over 12 weeks in insulin-resistant women with PCOS, identifying that in this context, rosiglitazone promoted ovulation and decreased IR and insulinemia in a dose-dependent manner, and that circulating insulin and testosterone were decreased in women able to ovulate [229]. The TZDs pioglitazone and rosiglitazone are used in PCOS treatment to alleviate hyperinsulinemia, androgen excess, and anovulation, but also have the undesirable effects of weight gain, oedema, and increased risk of cardiovascular disease [230]. TZD and other insulin sensitizers increase ovulation rates in women with PCOS [231]. In the adrenal gland, PPAR*γ* is expressed primarily in the zona glomerulosa [232]. In human adrenocortical carcinoma (H295R) cells, pioglitazone suppresses angiotensin II-induced aldosterone secretion and CYP11B2 expression [232]. In mice, the PPAR*γ agonist* rosiglitazone alleviates adrenal hypertrophy and hypercortisolism caused by N-acetylcysteine (NAC) therapy [233].

## 5. Interactions between PPARs and The ECS

**Figure 3:**
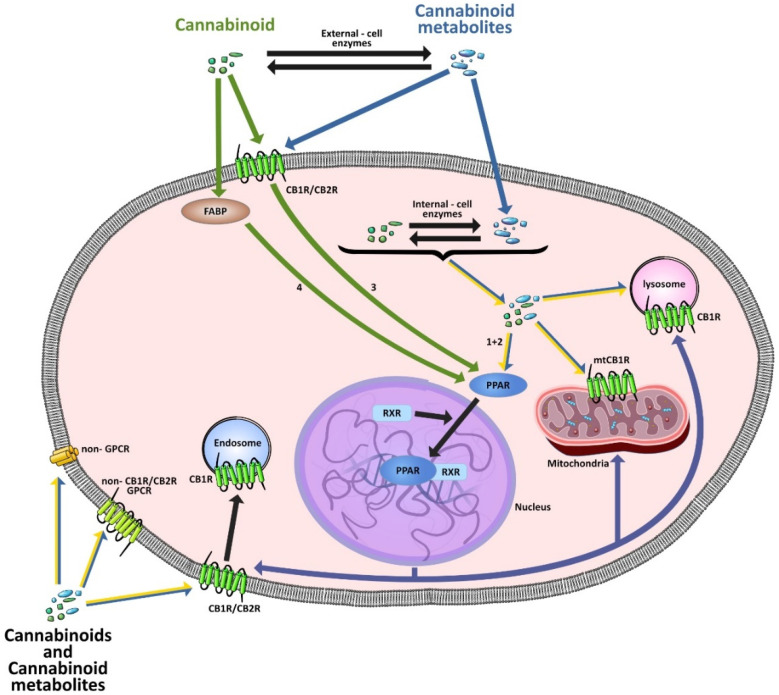
Interactions between PPARs and the ECS. The blue-yellow arrows (1, 2) and green arrows (3, 4) indicate the different mechanisms of action of cannabinoids on PPARs. Navy blue lines indicate PPAR modulation of *ECS* gene expression, such as receptors, enzymes, and transport proteins. The figure does not include the influence of PPARs on expression of enzymes such as cyclooxygenases, lipoxygenases, or FAAH for the sake of clarity. Abbreviations: CB1R, cannabinoid receptor type 1; CB2R, cannabinoid receptor type 2; mtCB1R, mitochondrial cannabinoid receptor type 1; FABPs, non-fatty acid-binding proteins; non-CB1R/CB2R GPCRs, G protein-coupled receptors other than CB1R or CB2R; non-GPCR, other receptors targeted by cannabinoids; PPAR, peroxisome proliferator-activated receptor; RXR, retinoid X receptor.

CBs regulate PPARs [234,235,236] via diverse mechanisms, including direct PPAR binding (1), PPAR activation by cannabinoid metabolites (2), indirect PPAR activation by downstream GPCR signaling cascades (3), and transport of CBs to PPARs via fatty acid-binding proteins (FABPs) (4) (Figure 3).

### 5.1. Cannabinoids and Their Metabolites as PPAR Ligands

Some CBs and their metabolites could potentially function as PPAR agonists. Specific studies are cited in Table 3.

Endocannabinoids and their metabolites can affect PPARs. AEA and 2-AG endocannabinoids activate both PPARα and PPARγ [237,238,239,240]. PEA can activate PPARα [241]. Oleoylethanolamide is a high-affinity PPARα agonist and activates the transcriptional activity of PPARβ/δ and PPARα [242]. Oleamide transactivates PPARα, PPARβ/δ, and PPARγ [243]. OEA, noladin ether, and virodhamine are PPARα ligands [237]. Esters derived from 2-AG affect PPAR receptors. 15d-PGJ2 glycerol ester activates PPARγ, and the 15-Hydroxyeicosatetraenoic acid glycerol ester increases the transcriptional activity of PPARα [239,244].

Plant CBs affect PPARs, especially the PPARγ receptor. Cannabidiol increases the transcriptional activity of PPARγ, which is inhibited by a selective PPARγ antagonist [245]. Further, Δ9-THC is a PPARγ ligand [246]. PPARγ is also activated by CBs such as Δ9-THC, CBC, CBD, and CBG [245,246,247]. Δ9-THCA is also a PPARγ agonist [248].

Synthetic CBs such as WIN 55212-2 bind and activate the transcriptional activities of PPARα and PPARγ [237,249]. The novel synthetic cannabinoid derivatives VCE-004.3, VCE-004.8, and VCE-0003 also function as dual PPARγ/CB2R agonists [247,250,251]. Interestingly, VCE-004.3 is also a CB1R antagonist [250]. The synthetic cannabinoid aulemic acid is a dual CB2R/PPARγ agonist [252,253].

Some CBs and their derivatives target PPARs but not CBRs. Oleylethanolamide does not bind CBRs but is a PPARα agonist [242]. Cannabidiolic acid is a dual PPARα/PPARγ agonist [254] but does not activate CB1R and is only a weak CB2R agonist [255]. Cannabimovone is a dual PPARα/PPARγ agonist [256] and activates TRPV1, but both are devoid of significant affinity for either CB1R or CB2R [257]. Cannabigerolic acid (CBGA) is a dual PPARα/PPARγ agonist [254] but has very low affinity for CB1R and CB2R [258].

Chromenpyrazoles have been synthesized as anticancer drugs with both cannabinoid antitumoral properties and quinone cytotoxicity. Chromenopyrazole 4 is both a CB1R and PPARγ agonist [259]. The amphetamine derivative OLHHA is a CB1R antagonist and PPARα agonist [260,261].

**Table 3:** PPAR activation by cannabinoids and cannabinoid metabolites.

Type of PPAR	Cannabinoid or Cannabinoid Metabolite	
**PPAR** **α**	WIN 55212-2 [237] noladin ether [237] virodhamine [237] 2-AG [239] AEA [237] 15-HETE glycerol ester [239] CBDA [254]	CBM [256] CBGA [254] PEA [241] OEA [237,242] ODA [243] OLHHA [260,261]
**PPAR** **β/δ**	OEA [242] ODA [243]	
**PPAR** **γ**	∆9-THC [246,247] CBC [247] CBD [245,247] CBG [247] 2-AG [238] AEA [240] 15d-PGJ2 glycerol ester [244] WIN 55212-2 [249] ∆9-THCA [248]	ODA [243] CBDA [254] CBM [256] CBGA [254] AJA [252,253] VCE-003 [247] VCE–004.3 [250] VCE–004.8 [251] chromenopyrazole 4 [259]

### 5.2. Link of Cannabinoid-Metabolizing Enzymes to PPARs

Inhibitors of the fatty acid amide hydrolase FAAH enzyme increase AEA and OEA levels [262]. Interestingly, PEA inhibits expression of FAAH, the primary enzyme responsible for AEA degradation [111]. Some cannabinoid metabolites produced by cyclooxygenase and lipoxygenase enzymes, which are also cannabinoid-metabolizing enzymes, function as PPARγ agonists. Moreover, PPARs regulate expression of lipooxygenase and cyklooxygenase. COX-2 and *PPARγ* gene expression are related in some contexts. In CaSki cells, COX-2 and *PPARγ* mRNA levels are inversely regulated by PPARγ ligands, which upregulate PPARγ but downregulate COX-2 [263]. Interestingly, a docking study demonstrated that some aryloxyacetic derivatives function as PPAR agonists and FAAH inhibitors [264].

### 5.3. Link between Cannabinoids Receptors and PPARs

A phylogenetic profiling study demonstrated that CB1R evolutionarily coevolves with PPARα [265]. The use of AM6545 to block peripheral CB1R in diet-induced obese mice alleviates liver steatosis and liver injury in WT but not *Pparα^-/-^* mice [265]. With this antisteatotic effect induced by AM6545 in WT diet-induced obese mice, a study demonstrated increased hepatic expression (Western blotting) and activity of PPARα (luciferase reporter assay) and increased hepatic OEA and PEA levels (liquid chromatography–tandem mass spectrometry (LC-MS/MS)), which can activate PPARα. In the liver, CB1R regulates p53 expression, acetylation, and transcriptional activity, increasing expression of miR-22, which specifically targets sirtuin 1 and PPARα [265].

The expressions of CBR1 and PPARγ are mutually regulated. In adipocytes, Win 55,212 upregulates PPARγ expression, but rosiglitazone significantly downregulates CBR1 [129]. CB1R activation also affects PPARγ and adiponectin expression. Chronic HU-210 activation of cannabinoid receptors stimulates expression of PPARγ, an early marker of adipocyte differentiation, and inhibits the expression of adiponectin, a late adipocyte differentiation marker [266].

In rat microglia cell culture, PEA enhances CB2R expression via PPARα activation [267]. In addition, bioinformatic analysis and chromatin immunoprecipitation studies indicate that CB2R is directly regulated by the PEA activation of PPARα [267].

Interestingly, prior studies using molecular biology methods have identified a PPARα-mediated pathway triggering TRPV1 channel activation and desensitization [268].

### 5.4. Non-Cannabinoid Dual Ligands of CBRs and PPARs

In this subchapter, we will discuss natural and synthetic compounds that are not CBs but function as CBR and PPAR ligands. Magnolol and honokiol are lignans derived from extracts of the Magnolia officinalis plant. Both compounds are agonists of CB1R, PPARα, and PPARγ, but have differential effects on CB2R. Magnolol is a CB2R agonist, but honokiol is a CB2R antagonist [269,270]. Beta-caryophyllene (BCP) is a terpene and has flavor-enhancing properties in plant foods and teas. It functions as a dual CB2R and PPARα agonist [271].

Fenofibrate is the most used fibrate in clinical practice. Interestingly, fenofibrate is a partial agonist of CB1R and a CB2R agonist [272]. Dual ligands have been designed and synthesized to target both PPARs and CBR2. Rimonabant fibrate 2 was generated by merging the pharmacophores of the fibrates (PPARα) and rimonabant diarylpyrazole (CB1R antagonist) and is a CB1R antagonist and PPARα agonist [273]. The action of non-cannabinoid dual ligands on CBRs and PPARs is summarized in Table 4.

## 6. Concluding Remarks and Future Directions

**Figure 4:**
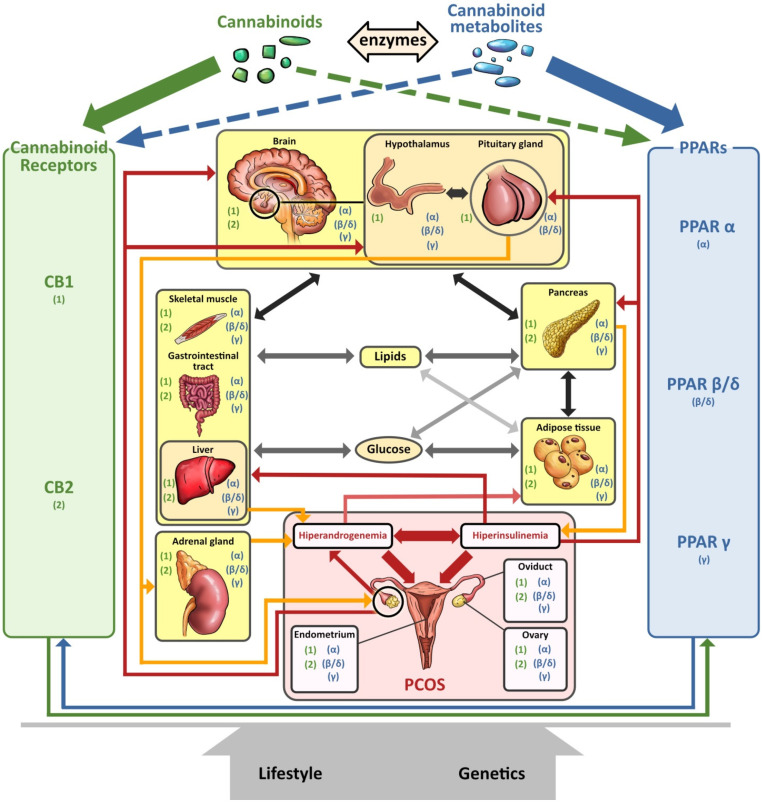
Pathophysiology of PCOS with presence of CBRs and PPARs in human tissues. Abbreviations: CB1Rm cannabinoid receptor type 1; CB2R, cannabinoid receptor type 2; PPAR, peroxisome proliferator-activated receptor.

We discuss the connection between the ECS (including CBs), PPARs, PCOS, obesity, and fertility. This is a novel axis in the pathophysiology of PCOS and uncovers novel potential therapeutic modalities. The pathophysiology of PCOS with CBRs and PPARs in human tissues is illustrated in Figure 4.

The primary commonality of these systems is carbohydrate and lipid metabolism. The ECS and PPARs are crucial regulators of energy metabolism and metabolic homeostasis and control processes such as hunger and satiety mechanisms in CNS. A robust body of literature has consistently documented that fertility disorders in PCOS are associated with obesity. Elements of the ECS and PPARs affect ovarian function and early stages of pregnancy. Additionally, mitochondrial dysfunction occurs in PCOS ovaries. Intracellularly, mtCB1R controls respiration and energy production, and PPARs modulate mitochondrial function. The effects of CBs are modulated primarily by CB1R and CB2R, but CBs also interact with other non-cannabinoid receptors such as TRPV1 or PPARs. Moreover, *CBRs* and PPARs are linked, as are cannabinoid-metabolizing enzymes and PPARs. Interestingly, some non-cannabinoids function as dual *CBRs*/PPAR agonists.

PPAR ligands—TZDs are one of several treatment options approved for PCOS. Another PPAR ligand—fibrates may be helpful in PCOS-related dyslipidemia as hypolipidemic drugs, but further research is needed. While PPAR ligands are approved for the management of PCOS, new and “old” ligands with various applications in the PCOS should still be investigated in the future. Unfortunately, there are a lack of reliable data on the use of cannabinoids as medications in PCOS. Cannabinoids have many ways of action in multiple cell types in the human body. They directly and indirectly affect the organs and imply a complex pathology of PCOS. Their use in PCOS treatment depends on the mechanism of action of the individual cell components. We suggest that CBs may play a role in treating PCOS by acting directly and indirectly on the cell through its cell membrane receptors, as well as inside the cell, e.g., the mitochondrial mtCB1R. An important aspect of treating PCOS is focused on managing PCOS disturbances. We have previously described how ECS and PPARs play an important role in cellular metabolism. We also pointed out the crucial roles of these systems in the metabolism of the entire organism. This is why we consider CBs and PPAR ligands to be important in treatment of overweight and obesity in PCOS women. We also suggest that CBs may play role in PCOS treatment by directly influencing endometrium, ovaries, or hormone system. Thus, different cannabinoid ligands are possible as PCOS medications, but further studies are necessary. The diversity of PCOS types leads us to suspect that they will permit individualization of future treatments. It is worth testing whether combinations of CBs and PPAR ligands can improve treatment efficiency.

There is a great potential to use CBs and their metabolites and non-cannabinoid dual CBRs/PPAR agonists as novel interventions for PCOS and related disorders. PCOS pathophysiology is complex and poorly understood. We demonstrate that the ECS and PPARs play an important role in the pathogenesis of PCOS (including mtCB1R). The complexity of the ECS and the PPARs will allow the development of diverse therapeutic modalities targeting these interrelated systems. Further, these interventions can be used to develop personalized approaches to treatment based on individual patient characteristics.

## Figures and Tables

**Table 1:** Percentage distribution of phenotypes in PCOS patients [30].

Phenotype	Includes	Definition	Distribution (%)
A	HA + OD + PCO	Full-blown syndrome PCOS	67.7%
B	HA + OD	Non-PCO PCOS	11%
C	HA + PCO	Ovulatory PCOS	17.7%
D	OD + PCO	Non-hyperandrogenic PCOS	3.6%

HA, hyperandrogenism; OD, ovulatory dysfunction = oligo-anovulation; PCO, polycystic ovarian morphology.

**Table 2:** Cannabinoids, canabimimetic molecules, cannabinoid-like molecules and derivatives, PPAR ligands, and non-cannabinoid dual ligands of CBRs and PPARs.

Endocannabinoids			
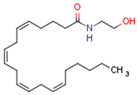 *AEA (Anandamide) (5Z,8Z,11Z,14Z)-N-(2-hydroxyethyl)icosa-5,8,11,14-tetraenamide C_22_H_37_NO_2_		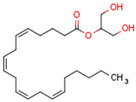 *2-AG (2-Arachidonoylglycerol) 1,3-dihydroxypropan-2-yl (5Z,8Z,11Z,14Z)-icosa-5,8,11,14-tetraenoate C_23_H_38_O_4_	
Endogenous cannabimimetic molecules and derivatives			
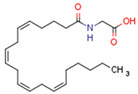 NAGLy (N-arachidonoylglycine) 2-[[(5Z,8Z,11Z,14Z)-icosa-5,8,11,14-tetraenoyl]amino]acetic acid C_22_H_35_NO_3_	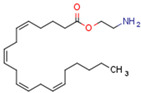 *O-AEA (O-arachidonoyl ethanolamine = virodhamine) 2-aminoethyl (5Z,8Z,11Z,14Z)-icosa-5,8,11,14-tetraenoate C_22_H_37_NO_2_	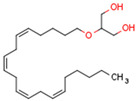 *2-AGE (2-Arachidonyl glyceryl ether = noladin ether = noladin) 2-[(5Z,8Z,11Z,14Z)-icosa-5,8,11,14-tetraenoxy]propane-1,3-diol C_23_H_40_O_3_	
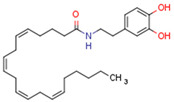 NADA (N-arachidonoyldopamine) (5Z,8Z,11Z,14Z)-N-[2-(3,4-dihydroxyphenyl)ethyl]icosa-5,8,11,14-tetraenamide C_28_H_41_NO_3_	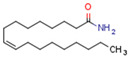 *ODA (Oleamide= Oleic acid amide) (Z)-octadec-9-enamide C_18_H_35_NO	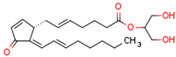 *15d-PGJ 2-glycerol ester (15-deoxy-delta12,14-prostaglandin J2-2-glycerol ester) 1,3-dihydroxypropan-2-yl (Z)-7-[(1S,5E)-5-[(Z)-oct-2-enylidene]-4-oxocyclopent-2-en-1-yl]hept-5-enoate C_23_H_34_O_5_	
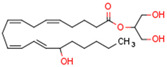 *15-HETE-glycerol ester (2-(15-Hydroxyeicosatetraenoyl)-sn-glycerol) 1,3-dihydroxypropan-2-yl (5Z,8Z,11Z,13E,15S)-15-hydroxyicosa-5,8,11,13-tetraenoate C_23_H_38_O_5_		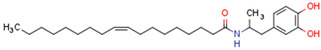 *OLHHA N-[2-(3,4-dihydroxyphenyl)-1-methylethyl]-9Z-octadecenamide C_27_H_45_NO_3_	
Endocannabinoid-like compounds			
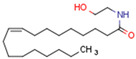 ***OEA (Oleoylethanolamine) (Z)-N-(2-hydroxyethyl)octadec-9-enamide C_20_H_39_NO_2_	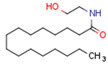 *PEA (Palmitoylethanolamide) N-(2-hydroxyethyl)hexadecanamide C_18_H_37_NO_2_	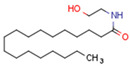 SEA (Stearoylethanolamide) N-(2-hydroxyethyl)octadecanamide C_20_H_41_NO_2_	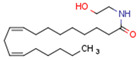 LEA (Linoleoylethanolamide) (9Z,12Z)-N-(2-hydroxyethyl)octadeca-9,12-dienamide C_20_H_37_NO_2_
Phytocannabinoids			
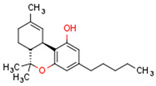 *Δ9-THC (delta9-Tetrahydrocannabinol =Dronabinol) (6aR,10aR)-6,6,9-trimethyl-3-pentyl-6a,7,8,10a-tetrahydrobenzo[c]chromen-1-ol C_21_H_30_O_2_	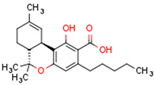 *Δ9-THCA (delta(9)-Tetrahydrocannabinolic acid) (6aR,10aR)-1-hydroxy-6,6,9-trimethyl-3-pentyl-6a,7,8,10a-tetrahydrobenzo[c]chromene-2-carboxylic acid C_22_H_30_O_4_	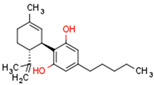 *CBD (Cannabidiol) 2-[(1R,6R)-3-methyl-6-prop-1-en-2-ylcyclohex-2-en-1-yl]-5-pentylbenzene-1,3-diol C_21_H_30_O_2_	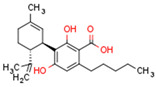 ***CBDA (Cannabidiolic acid) 2,4-dihydroxy-3-[(1R,6R)-3-methyl-6-prop-1-en-2-ylcyclohex-2-en-1-yl]-6-pentylbenzoic acid C_22_H_30_O_4_
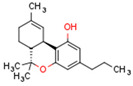 Δ9-THCV (delta(9)-Tetrahydrocannabivarin (6aR,10aR)-6,6,9-trimethyl-3-propyl-6a,7,8,10a-tetrahydrobenzo[c]chromen-1-ol C_19_H_26_O_2_	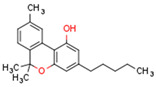 CBN (Cannabinol) 6,6,9-trimethyl-3-pentylbenzo[c]chromen-1-ol C_21_H_26_O_2_	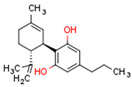 CBDV (Cannabidivarin = GWP42006) 2-[(1R,6R)-3-methyl-6-prop-1-en-2-ylcyclohex-2-en-1-yl]-5-propylbenzene-1,3-diol C_19_H_26_O_2_	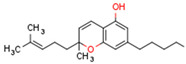 *CBC (Cannabichrome) 2-methyl-2-(4-methylpent-3-enyl)-7-pentylchromen-5-ol C_21_H_30_O_2_
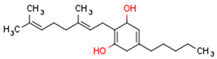 *CBG (Cannabigerol) 2-[(2E)-3,7-dimethylocta-2,6-dienyl]-5-pentylbenzene-1,3-diol C_21_H_32_O_2_	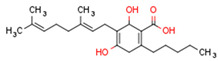 ***CBGA (Cannabigerolic acid) 3-[(2E)-3,7-dimethylocta-2,6-dienyl]-2,4-dihydroxy-6-pentylbenzoic acid C_22_H_32_O_4_	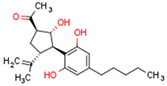 ***CBM (Cannabimovone) 1-[(1R,2R,3R,4R)-3-(2,6-dihydroxy-4-pentylphenyl)-2-hydroxy-4-prop-1-en-2-ylcyclopentyl]ethanone C_21_H_30_O_4_	
Phytocannabinoid derivatives			
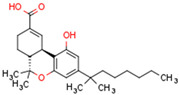 *AJA (Ajulemic acid) (6aR,10aR)-1-hydroxy-6,6-dimethyl-3-(2-methyloctan-2-yl)-6a,7,10,10a-tetrahydrobenzo[c]chromene-9-carboxylic acid C_25_H_36_O_4_	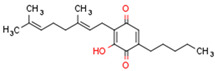 *CBGQ =VCE003 (Cannabigeroquinone) 2-[(2E)-3,7-dimethylocta-2,6-dienyl]-3-hydroxy-5-pentylcyclohexa-2,5-diene-1,4-dione C_21_H_30_O_3_	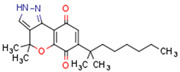 *Chromenopyrazoledione 4 7-(1′,1′-dimethylheptyl)-1,4-dihydro-4,4-dimethylchromen [4,3-c]pyrazol-6,9-dione C_21_H_28_N_2_O_3_	
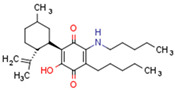 *VCE-004.3 (1′R,6′R)-6-hydroxy-3′-methyl-4-pentyl-3-(pentylamino)-6′-(prop-1-en-2-yl)-[1,1′-bi(cyclohexane)]-2′,3,6-triene-2,5-dione C_26_H_39_NO_3_		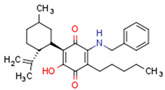 *VCE-004.8 5-(benzylamino)-4-hydroxy-3-[(1R,6R)-3-methyl-6-prop-1-en-2-ylcyclohex-2-en-1-yl]-6-pentylcyclohexa-3,5-diene-1,2-dione C_28_H_35_NO_3_	
Synthetic cannabinoids			
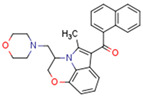 *WIN 55,212 [(11R)-2-methyl-11-(morpholin-4-ylmethyl)-9-oxa-1-azatricyclo [6.3.1.04,12]dodeca-2,4(12),5,7-tetraen-3-yl]-naphthalen-1-ylmethanone C_27_H_26_N_2_O_3_		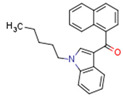 JWH 018 naphthalen-1-yl-(1-pentylindol-3-yl)methanone C_24_H_23_NO	
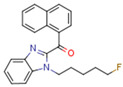 AM 2201 [1-(5-fluoropentyl)indol-3-yl]-naphthalen-1-ylmethanone C_24_H_22_FNO	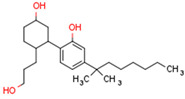 CP 55940 2-[(1R,2R,5R)-5-hydroxy-2-(3-hydroxypropyl)cyclohexyl]-5-(2-methyloctan-2-yl)phenol C_24_H_40_O_3_	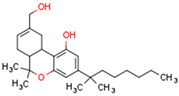 HU 210 (6aR,10aR)-9-(hydroxymethyl)-6,6-dimethyl-3-(2-methyloctan-2-yl)-6a,7,10,10a-tetrahydrobenzo[c]chromen-1-ol C_25_H_38_O_3_	
Endogenous PPAR ligands			
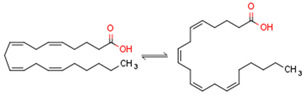 AA (Arachidonic acid) (5Z,8Z,11Z,14Z)-icosa-5,8,11,14-tetraenoic acid C_20_H_32_O_2_	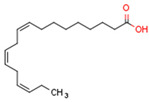 ALA (alpha-Linolenic acid) (9Z,12Z,15Z)-octadeca-9,12,15-trienoic acidC_18_H_30_O_2_	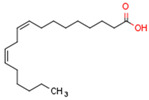 LA (Linolic acid = linoleic acid) (9Z,12Z)-octadeca-9,12-dienoic acid C_18_H_32_O_2_	
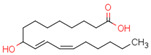 9-HODE (9-Hydroxyoctadecadienoic acid,) (10E,12Z)-9-hydroxyoctadeca-10,12-dienoic acid C_18_H_32_O_3_	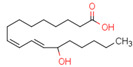 13-HODE (13-Hydroxyoctadecadienoic acid) (9E,11E)-13-hydroxyoctadeca-9,11-dienoic acid C_18_H_32_O_3_	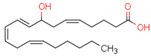 8-HETE (8-Hydroxyeicosatetraenoic acid) (5Z,9E,11Z,14Z)-8-hydroxyicosa-5,9,11,14-tetraenoic acid C_20_H_32_O_3_	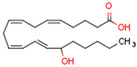 15-HETE (15-Hydroxyeicosatetraenoic acid) (5E,8Z,11Z,13Z)-15-hydroxyicosa-5,8,11,13-tetraenoic acid C_20_H_32_O_3_
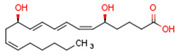 LTB4 (Leukotriene B4) (5S,6Z,8E,10E,12R,14Z)-5,12-dihydroxyicosa-6,8,10,14-tetraenoic acid C_20_H_32_O_4_	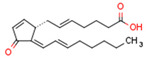 15d-PGJ 2 (15-deoxy-delta12,14-prostaglandin J2) (Z)-7-[(1S,5E)-5-[(E)-oct-2-enylidene]-4-oxocyclopent-2-en-1-yl]hept-5-enoic acid C_20_H_28_O_3_	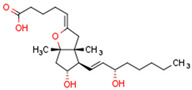 PGI 2 (Prostaglandin I2 = Epoprostenol = Prostacyclin) (5Z)-5-[(3aR,4R,5R,6aS)-5-hydroxy-4-[(E,3S)-3-hydroxyoct-1-enyl]-3,3a,4,5,6,6a-hexahydrocyclopenta[b]furan-2-ylidene]pentanoic acid C_20_H_32_O_5_	
Fibrates			
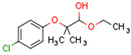 Clofibrate ethyl 2-(4-chlorophenoxy)-2-methylpropanoate C_12_H_15_ClO_3_	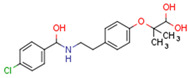 Bezafibrate 2-[4-[2-[(4-chlorobenzoyl)amino]ethyl]phenoxy]-2-methylpropanoic acid C_19_H_20_ClNO_4_	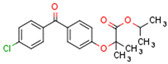 *****Fenofibrate propan-2-yl 2-[4-(4-chlorobenzoyl)phenoxy]-2-methylpropanoate C_20_H_21_ClO_4_	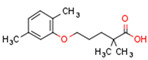 Gemfibrozil 5-(2,5-dimethylphenoxy)-2,2-dimethylpentanoic acid C_15_H_22_O_3_
Synthetic PPAR β/δ agonists			
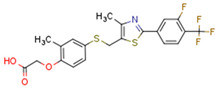 GW 0742 2-[4-[[2-[3-fluoro-4-(trifluoromethyl)phenyl]-4-methyl-1,3-thiazol-5-yl]methylsulfanyl]-2-methylphenoxy]acetic acid C_21_H_17_F_4_NO_3_S_2_		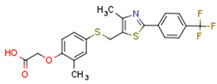 GW 501516 (Endurobol) 2-[2-methyl-4-[[4-methyl-2-[4-(trifluoromethyl)phenyl]-1,3-thiazol-5-yl]methylsulfanyl]phenoxy]acetic acid C_21_H_18_F_3_NO_3_S_2_	
Thiazolidinediones			
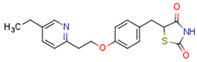 Pioglitazone 5-[[4-[2-(5-ethylpyridin-2-yl)ethoxy]phenyl]methyl]-1,3-thiazolidine-2,4-dione C_19_H_20_N_2_O_3_S	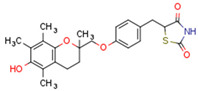 Troglitazone 5-[[4-[(6-hydroxy-2,5,7,8-tetramethyl-3,4-dihydrochromen-2-yl)methoxy]phenyl]methyl]-1,3-thiazolidine-2,4-dione C_24_H_27_NO_5_S	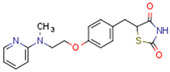 Rosiglitazone 5-[[4-[2-[methyl(pyridin-2-yl)amino]ethoxy]phenyl]methyl]-1,3-thiazolidine-2,4-dione C_18_H_19_N_3_O_3_S	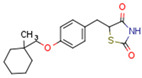 Ciglitazone 5-[[4-[(1-methylcyclohexyl)methoxy]phenyl]methyl]-1,3-thiazolidine-2,4-dione C_18_H_23_NO_3_S
Other non-cannabinoid dual ligands of CBRs and PPARs			
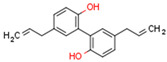 *****Magnolol 2-(2-hydroxy-5-prop-2-enylphenyl)-4-prop-2-enylphenol C_18_H_18_O_2_	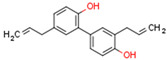 *****Honokiol 2-(4-hydroxy-3-prop-2-enylphenyl)-4-prop-2-enylphenol C_18_H_18_O_2_	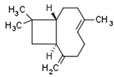 *****BCP (beta-Caryophyllene) (1R,4E,9S)-4,11,11-trimethyl-8-methylidenebicyclo [7.2.0]undec-4-eneC_15_H_24_	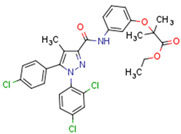 *****Rimonabant fibrate
Other			
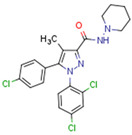 SR141716 (Rimonabant) 5-(4-chlorophenyl)-1-(2,4-dichlorophenyl)-4-methyl-N-piperidin-1-ylpyrazole-3-carboxamide C_22_H_21_Cl_3_N_4_O			

*—cannabinoids, cannabinoid-like compounds, cannabimimetic molecules and derivatives that activate PPARs. ***—cannabinoids, cannabinoid-like compounds that activate PPARs but not CBRs. *****—non-cannabinoid dual ligands of CBRs and PPARs.

**Table 4:** Non-cannabinoid dual ligands of CBRs and PPARs.

Ligand	CBRs		PPARs	
	CB1R	CB2R	PPARα	PPARγ
Magnolol [269,270]	(+) agonist	(+) agonist	(+) agonist	(+) agonist
Honokiol [269,270]	(+) agonist	(−) antagonist	(+) agonist	(+) agonist
BCP [271]		(+) agonist	(+) agonist	
Fenofibrate [272]	(+) agonist	(+) agonist	(+) agonist	
Rimonabant fibrate 2 [273]	(−) antagonist		(+) agonist	

## Data Availability

Not applicable.
